# Modified approach to the characterization of adrenal nodules using a standard abdominal magnetic resonance imaging protocol

**DOI:** 10.1590/0100-3984.2015.0102

**Published:** 2017

**Authors:** António P. Matos, Richard C. Semelka, Vasco Herédia, Mamdoh AlObaidiy, Filipe Veloso Gomes, Miguel Ramalho

**Affiliations:** 1 Department of Radiology, University of North Carolina at Chapel Hill, Chapel Hill, NC, USA, and Hospital Garcia de Orta, Almada, Portugal.; 2 Department of Radiology, University of North Carolina at Chapel Hill, Chapel Hill, NC, USA.; 3 Department of Radiology, Hospital do Espírito Santo, Évora, Portugal.; 4 Department of Radiology, University of North Carolina at Chapel Hill, Chapel Hill, NC, USA, and King Faisal Specialist Hospital and Research Center, Riyadh, Saudi Arabia.; 5 Department of Radiology, Hospital de Faro, Portugal.

**Keywords:** Adenoma, Adrenal gland neoplasms, Image enhancement, Contrast media, Magnetic resonance imaging

## Abstract

**Objective:**

To describe a modified approach to the evaluation of adrenal nodules using a standard abdominal magnetic resonance imaging protocol.

**Materials and Methods:**

Our sample comprised 149 subjects (collectively presenting with 132 adenomas and 40 nonadenomas). The adrenal signal intensity index was calculated. Lesions were grouped by pattern of enhancement (PE), according to the phase during which the wash-in peaked: arterial phase (type 1 PE); portal venous phase (type 2 PE); and interstitial phase (type 3 PE). The relative and absolute wash-out values were calculated. To test for mean differences between adenomas and nonadenomas, Student's *t*-tests were used. Receiver operating characteristic curve analysis was also performed.

**Results:**

The mean adrenal signal intensity index was significantly higher for the adenomas than for the nonadenomas (*p* < 0.0001). Chemical shift imaging showed a sensitivity and specificity of 94.4% and 100%, respectively, for differentiating adenomas from nonadenomas. Of the adenomas, 47.6%, 48.5%, and 3.9%, respectively, exhibited type 1, 2, and 3 PEs. For the mean wash-in proportions, significant differences were found among the enhancement patterns. The wash-out calculations revealed a trend toward better lesion differentiation for lesions exhibiting a type 1 PE, showing a sensitivity and specificity of 71.4% and 80.0%, respectively, when the absolute values were referenced, as well as for lesions exhibiting a type 2 PE, showing a sensitivity and specificity of 68.0% and 100%, respectively, when the relative values were referenced. The calculated probability of a lipid-poor lesion that exhibited a type 3 PE being a nonadenoma was > 99%.

**Conclusion:**

Subgrouping dynamic enhancement patterns yields high diagnostic accuracy in differentiating adenomas from nonadenomas.

## Resumo

**Objetivo:**

Descrever uma abordagem modificada para a avaliação de nódulos adrenais utilizando um protocolo padrão de ressonância magnética abdominal.

**Materiais e Métodos:**

A nossa amostra foi composta de 149 indivíduos (132 adenomas e 40 não adenomas). O índice de intensidade do sinal adrenal foi calculado. As lesões foram agrupadas em três grupos de acordo com o tempo de *wash-in* até atingir o pico de realce (arterial, tipo 1 PE; portal-venoso, tipo 2 PE; ou intersticial, tipo 3 PE). O *wash-out* relativo e o absoluto foram calculados. O teste t de Student foi utilizado para examinar diferenças entre adenomas e não adenomas. A análise da curva ROC foi realizada.

**Resultados:**

O índice de intensidade médio do sinal adrenal dos adenomas adrenais foi significativamente maior (*p* < 0,0001). A imagem de fora-de-fase teve sensibilidade de 94,4% e especificidade de 100% para diferenciar adenomas de não adenomas. Do total de adenomas, 47,6% exibiram tipo 1 PE, 48,5% tipo 2 PE e 3,9% tipo 3 PE. Para todos os padrões de realce foram encontradas diferenças significativas para as porcentagens médias de *wash-out*. Houve tendência para uma melhor diferenciação da lesão utilizando o cálculo absoluto de *wash-out* para lesões apresentando tipo 1 PE e *wash-out* relativo para lesões apresentando tipo 2 PE, com sensibilidade e especificidade de 71,4% e 80% e 68% e 100%, respectivamente. A probabilidade calculada de uma lesão pobre em lipídios exibir um padrão tipo 3 PE de ser um não adenoma foi superior a 99%.

**Conclusão:**

O agrupamento de padrões dinâmicos de realce proporciona elevada precisão diagnóstica na diferenciação de adenomas de não adenomas adrenais.

## INTRODUCTION

Adrenal nodules are frequently encountered in daily clinical practice^(1,2)^. Magnetic resonance imaging (MRI) is an established method for the evaluation of adrenal lesions. Chemical-shift imaging (CSI) is an intracellular, lipid-sensitive technique that has become the mainstay of MRI for the evaluation of solid adrenal lesions^(3-6)^. CSI has high sensitivity and specificity in the differentiation between malignant and benign lesions, because benign lesions frequently have higher lipid content in patients without a history of fatcontaining primary tumors^(4,7)^. Although the same rationale has been utilized for unenhanced computed tomography (CT), MRI appears to be more sensitive for the detection of low levels of intracytoplasmic lipids^(8,9)^. Nevertheless, 10-20% of benign adenomas are lipid-poor, and there is substantial overlap between benign and malignant lesions in terms of their appearance. Conversely, there are other lesions (benign and malignant) that exhibit high lipid content and can mimic lipid-rich adenomas^(10,11)^.

Several studies have suggested alternative strategies for facilitating the characterization of adrenal masses on MRI. Similar to CT, delayed contrast-enhanced imaging can be helpful in making that distinction^(12-14)^. However, the use of contrast wash-out patterns on delayed images may not be practical in MRI because of the long examination times required, including an additional 15-min delayed acquisition.

Some authors have shown the importance of early dynamic post-gadolinium contrast evaluation. Many adrenal adenomas, irrespective of their lipid-content, have an immediate homogenous capillary blush and rapid fading^(14,15)^, features not observed for the majority of malignant lesions. Other investigators have qualitatively or quantitatively evaluated the dynamic behavior of adrenal lesions in post-gadolinium studies^(16,17)^, which allows a more refined differential diagnosis.

The purpose of our study was to describe a modified approach for evaluating adrenal nodules using a standard abdominal MRI protocol, by categorizing adrenal lesion on dynamic post-gadolinium acquisitions and integrating these results with CSI.

## MATERIALS AND METHODS

**Figure 1:**
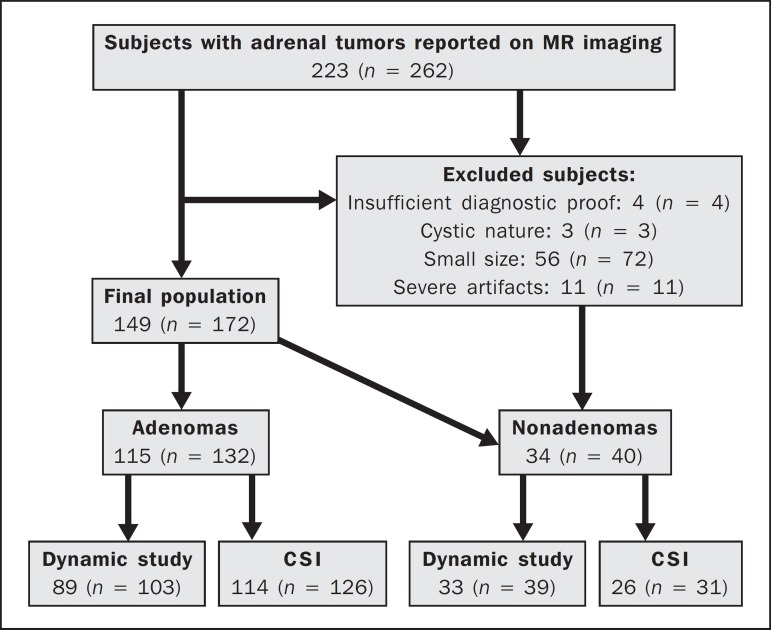
Flowchart providing information about the method of patient recruitment and the number of patients who underwent chemical-shift imaging and dynamic contrast-enhanced evaluation. Data in parentheses indicate the number of lesions.

Our institutional review board approved this observational retrospective study. We searched our institution's MRI reports database for cases of focal adrenal lesions treated between January 2008 and December 2012. We identified a total of 262 lesions in 223 consecutive subjects. Seventy-four subjects were excluded: because there was not sufficient clinical/histological data to allow lesion characterization (*n* = 4); because the lesion was homogeneously cystic (*n* = 3); because the transverse or anteroposterior maximum diameter of the lesion was less than 1 cm, which could result in partial volume effects (*n* = 56); or because severe imaging artifacts were present (*n* = 11). Therefore, the final sample comprised 149 subjects (86 females and 63 males; mean age, 65 ± 13 years) collectively presenting 172 lesions (Figure 1). Of those 172 lesions, 132 (in 115 subjects) were categorized as adenomas: because the pathology report was consistent with the diagnosis (*n* = 2); because the follow-up findings showed that the lesion remained stable for more than 6 months, with less than 10% variation in the maximum transverse diameter compared with previous multidetector CT or MRI scans (*n* = 2); or because the lesion had an adrenal signal intensity index (ASII) higher than 16.5%, there was no clinical or imaging evidence of extra-adrenal primary neoplasia, and there was no clinical or biochemical suspicion of pheochromocytoma (*n* = 128)^(6)^. Adenomas were further classified as lipid-rich if the ASII was greater than 16.5%^(4)^. A total of 40 lesions (in 34 subjects) were categorized as nonadenomas: 24 (in 18 subjects) were metastases; 7 (in as many subjects) were myelolipomas; 6 (in as many subjects) were pheochromocytomas; 2 (in as many subjects) were adrenal carcinomas; and 1 was an adrenal oncocytoma. The diagnosis of metastasis was established in subjects with a history of neoplasia, together with a *de novo* adrenal finding (*n* = 7), growth (more than 10% variation in size) on followup imaging (*n* = 13), a response to chemotherapy (*n* = 2), or histopathological confirmation (*n* = 2). The sites of the primary neoplasms are shown in Table 1.

**Table 1:** Primary origin of metastases.

Site/type of lesion	N	(n)
Lung		
Small cell carcinoma	1	(3)
Adenocarcinoma	2	(3)
Squamous adenocarcinoma	1	(1)
Undifferentiated non-small cell carcinoma	1	(1)
Colon adenocarcinoma	2	(3)
Renal cell carcinoma (clear cell subtype)	2	(3)
Hepatocellular carcinoma	1	(1)
Stomach adenocarcinoma	1	(1)
Cholangiocarcinoma	1	(1)
Melanoma	1	(1)
Uterine leiomyosarcoma	1	(1)
Liposarcoma	1	(1)
Non-Hodgkin lymphoma	1	(1)
Adenocarcinoma of unknown origin	2	(3)

*N*, number of patients; (*n*), number of lesions.

The seven myelolipomas were so classified because their fat content approached that of adjacent retroperitoneal fat, as shown on T1- and T2-weighted images with and without fat suppression. The myelolipomas were excluded from the ASII analysis and were included only in the dynamic postcontrast evaluation. Five of the six pheochromocytomas were surgically removed. In one subject, the diagnosis of pheochromocytoma was based on the typical clinical presentation and an elevated plasma level of metanephrine^(18)^. The two subjects with adrenal carcinomas had unilateral adrenal lesions and underwent surgery. The adrenal oncocytoma was diagnosed histologically following an adrenalectomy.

Of the 172 lesions in the final sample, 157 (126 adenomas and 31 nonadenomas) were considered in the CSI analysis. We excluded seven myelolipomas, one adenoma, and two metastases of renal cell carcinoma, the last because of severe image artifacts on CSI sequences.

In the dynamic post-contrast analysis, we included 142 lesions: 103 adenomas and 39 nonadenomas. Twenty-nine adenomas were excluded for the following reasons: severe images artifacts (*n* = 3); non-use of intravenous gadolinium (*n* = 17); and suboptimal arterial phase of imaging (*n* = 9). One metastasis (uterine leiomyosarcoma) was excluded due to the absence of dynamic evaluation.

### MRI technique

All subjects underwent MRI of the abdomen with a 1.5-T system (GE-Signa HDx; GE Healthcare, Waukesha, WI, USA) using a phased-array torso coil.

As part of our standard abdominal MRI protocol, the following sequences were performed: axial unenhanced twodimensional (2D) gradient-echo T1-weighted dual-echo inphase (repetition time/echo time [TR/TE] of 125/4.3 ms, 80° flip angle) and out-of-phase (125/2.1, 80° flip angle); coronal half-Fourier single-shot fast spin-echo T2-weighted (TR/TE of 1894.7/83.9 ms); axial half-Fourier single-shot fast spin-echo T2-weighted (TR/TE of 1800/89.9 ms), with and without fat suppression; and axial pre- and post-gadolinium fat-suppressed 3D gradient-echo (TR/TE of 4.17/1.98 ms, 12° flip angle) during the late arterial (also known as the hepatic arterial-dominant), portal venous, and interstitial phases^(19)^.

In all subjects, the contrast agent gadoterate meglumine (Dotarem; Guerbet, Paris, France) was administered intravenously, with an automated power-injector (Medrad, Pittsburgh, PA, USA), as a bolus of 0.1 mmol/kg at 2 mL/s, followed by a bolus of 20 mL saline flush. The arterial postcontrast sequence was acquired 20 s after the initiation of contrast injection, compared with 60-80 s after for the portal venous sequence and 3-4 min for the interstitial/equilibrium sequences.

### Image analysis

All quantitative measurements were performed by a single reader with eight years of experience in reading MRI scans. For each lesion, we recorded the largest diameter in the axial plane. For each lesion, the signal intensity (SI) was obtained in all sequences from region-of-interest (ROI) measurements at the same locations and at the same level. The ROI measurements were taken in a homogeneous areas devoid of vessels, cystic/necrotic tissue, and artifacts. ROIs were placed on each adrenal lesion to cover as much of the mass as possible, avoiding the edges of the lesion^(6)^. To maintain the consistency of the ROI sampling among the series, the reader used a copy and paste feature available on the workstation employed. Occasionally, minor adjustments were needed to adjust the location, but not the area, of the ROI.

The ASII was calculated according to the following formula^(4)^:

$$\left[\left({\mathrm{SI}}_{\mathrm{in}-\mathrm{phase}}-{\mathrm{SI}}_{\mathrm{out}-\mathrm{of}-\mathrm{phase}}\right){/SI}_{\mathrm{in}-\mathrm{phase}}\right]*100\%$$

For the dynamic post-contrast evaluation, the lesions were clustered in three groups, according to the pattern of enhancement (PE), based on the phase in which the enhancement peaked. Type 1 PE was assigned to a lesion if the washin peaked in the arterial phase and wash-out occurred during the subsequent phases. Type 2 PE was assigned to a lesion if the wash-in peaked in the portal venous phase and wash-out occurred during the interstitial phase. Type 3 PE was assigned to a lesion if the enhancement was steady and progressive throughout all phases, peaking in the interstitial phase.

The modified relative wash-out and absolute wash-out were quantified for type 1 PE and type 2 PE lesions. The following formulas were used:

WOr−type1PE=a−i/aWOa−type1PE=a−i/a−uWOr−type2PE=p−i/aWOa−type2PE=p−i/p−u

where *WO_r_* is the modified relative wash-out value; *WO_a_* is the absolute wash-out value; *a, i*, and *p* are the SI values in the arterial, interstitial, and portal venous phases of enhancement, respectively; and u is the SI value for an unenhanced scan. The proportional wash-in was calculated as follows:

WI=(x−u)/x*100

where *WI* is the wash-in value; *x* is the SI value for a given phase (arterial, portal venous, or interstitial); and *u* is the SI value for an unenhanced scan.

### Statistical analysis

Student's *t*-tests for independent-samples were used in order to test for mean differences between adenomas and nonadenomas in terms of lesion diameters and maximum wash-in percentage, as well as the relative and absolute washout rates. An F test for equal variances was used, and if *p* < 0.05, a Welch test assuming unequal variances was performed. *P* values were not adjusted for multiple comparisons. The probability of type 1, 2, and 3 PE lesions being adenomas was calculated by means of multiple event probability, as was that of type 1, 2, and 3 PE lesions being lipid-poor adenomas. The latter was calculated for the prevalence in our sample and for a hypothetical prevalence of 20% in the general population^(3,6,7,12)^.

Receiving operating characteristic (ROC) curve analysis was performed. An optimal cut-off value to differentiate adenomas from other adrenal tumors was calculated. That was defined as the value that produced the highest sum of sensitivity and specificity in the identification of adenoma. In all cases, values of *p* < 0.05 were considered to represent a statistically significant difference.

All statistical analyses were performed with MedCalc software for Windows, version 11.3.0.0 (MedCalc Software, Mariakerke, Belgium).

## RESULTS

The mean diameter of the adenomas and nonadenomas was 24.4 ± 8.2 mm (range, 13-58 mm) and 41.1 ± 24.4 mm (range, 13.8-109 mm), respectively, and the difference was statistically significant (*p* < 0.001).

The ROC analysis showed that a lesion diameter cut-off of 24.8 mm produced a sensitivity and specificity of 70% and 63.6%, respectively (area under the curve [AUC] = 0.729; 95% confidence interval [95% CI]: 0.628-0.831).

The mean ASII of the adrenal adenomas was significantly higher than was that of the nonadenomas (57.86 ± 20.96 vs. 2.60 ± 8.23; *p* < 0.0001). Of the 127 adenomas evaluated, 4 were considered lipid-poor (ASII ≤ 16.5%). Of the 40 nonadenomas, only one (a metastasis of liposarcoma) met the criteria for a lipid-rich adrenal nodule, with an ASII of 20.4%.

The ROC analysis showed that an ASII cut-off of 20.4% produced a sensitivity and specificity of 94.4% and 100%, respectively (AUC = 0.975; 95% CI: 0.936-0.993). Using the classic cut-off of 16.5%, the sensitivity and specificity were both 96.8%.

**Figure 2:**
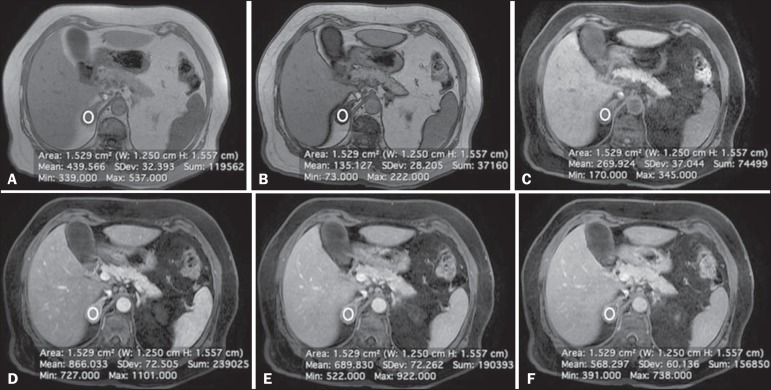
Lipid-rich adenoma in a 66-year-old woman. Transverse, T1-weighted in-phase (**A**, TR/TE of 125/4.3 ms, 80° flip angle) and out-of-phase (**B**, TR/TE of 125/2.1 ms, 80° flip angle) images of a right adrenal nodule that showed a signal drop in the out-of-phase sequence. The adrenal index was 69%. Transverse, T1-weighted three-dimensional gradient-echo MRI images with fat suppression (TR/TE of 4.17/1.98 ms, 12° flip angle), obtained before (**C**) and after gadolinium enhancement in the arterial (**D**), portal venous (**E**), and interstitial (**F**) phases, demonstrating a type 1 PE with a peak of enhancement in the arterial phase.

**Figure 3:**
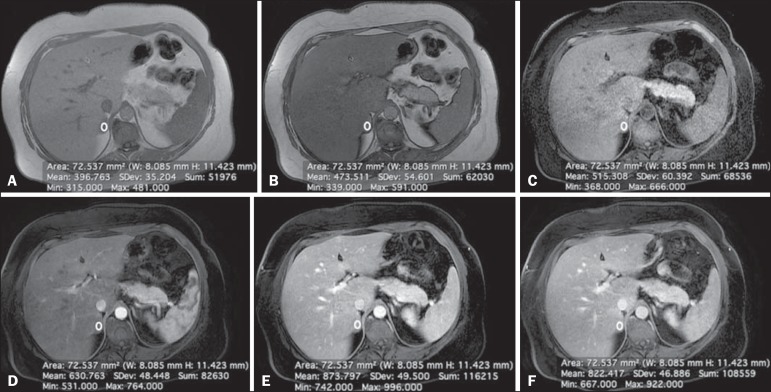
Lipid-poor adenoma in a 46-year-old woman. Transverse, T1 weighted in-phase (**A**, TR/TE of 125/4.3 ms, 80° flip angle) and out-of-phase (**B**, TR/TE of 125/2.1 ms, 80° flip angle) images of a well-circumscribed right adrenal nodule that did not show a signal drop in the out-of-phase sequence. The adrenal index was -19.3%. Transverse, fat-suppressed T1-weighted three-dimensional gradient-echo MRI images with fat suppression (TR/TE of 4.17/1.98 ms, 12° flip angle) obtained before (**C**) and after gadolinium enhancement in the arterial (**D**), portal venous (**E**), and interstitial (**F**) phases, demonstrating a type 2 PE with a peak of enhancement in the portal venous phase. The lesion presented long-term stability, which is consistent with a lipid-poor adrenal adenoma.

Figure 2 shows an adenoma in which the PE was categorized as type 1. Among the 127 adenomas evaluated, the PE was categorized as type 1 in 49 (47.6%), type 2 in 50 (48.5%), and type 3 in 4 (3.9%). The probability of type 1, 2, and 3 PE lesions being adenomas was 90.9%, 77% and 17.4%, respectively. Although the prevalence of lipid-poor adenomas in our study was low (3.2%), the probability of type 1, 2, and 3 PE lesions being lipid-poor adenomas was 1.9%, 2.0%, and 0.2%, respectively (Figure 3). Taking into consideration the fact that the reported prevalence of lipid-poor adenomas identified by CT in the literature is 20%^(3,6,7,12)^ and utilizing the calculated PEs described above, we found that the probability would be 9.6%, 9.8%, and 0.8% for type 1, 2, and 3 PE lesions, respectively. Among the 40 nonadenomas evaluated, the PE was categorized as type 1 in 5 (12.8%), type 2 in 15 (38.5%), and type 3 in 19 (48.7%).

For all PEs, significant differences for mean wash-in percentages were found between adenomas and nonadenomas (Table 2). The wash-in magnitude was significantly greater for adenomas with a type 2 or type 3 PE, as well as for nonadenomas with a type 1 PE, although there was substantial overlap between adenomas and nonadenomas.

**Table 2:** Quantitative data according to the enhancement pattern.

	Type 1 PE				Type 2 PE				Type 3 PE		
Evaluated parameters	Adenomas	Nonadenomas	*p*-value		Adenomas	Nonadenomas	*p*-value		Adenomas	Nonadenomas	*p*-value
Wash-in percentage	183.35 ± 55.82	240.12 ± 101.05	0.025		169.08 ± 43.56	110.52 ± 55.12	< 0.0001		157.76 ± 65.67	96.09 ± 47.98	0.0195
Absolute wash-out	42.28 ± 12.51	34.60 ± 8.41	0.1871		27.74 ± 11.06	18.50 ± 13.35	0.0088		Non-applicable	Non-applicable	
Relative wash-out	26.94 ± 8.29	24.05 ± 7.98	0.4613		16.85 ± 6.28	8.41 ± 4.97	< 0.0001		Non-applicable	Non-applicable	
Enhancement pattern	49 (47.6%)	5 (12.8%)			50 (48.5%)	15 (38.5%)			4 (3,9%)	19 (48.7%)	

*Note:* Data are mean ± standard deviation.

The results of the ROC curve analyses of relative and absolute wash-out values for type 1 and type 2 PE lesions are shown in Table 3. For lesions presenting with a type 1 PE, there was a trend toward better results in differentiating adenomas from nonadenomas when we used an absolute washout cut-off value of 38.4%, which produced a sensitivity and specificity of 71.4% and 80%, respectively (AUC = 0.735; 95% CI: 0.597-0.846). Conversely, for lesions presenting with a type 2 PE, the use of a relative wash-out cut-off value of 13.9% provided the best results, with a sensitivity and specificity of 68% and 100%, respectively (AUC = 0.857; 95% CI: 0.748-0.932). Using the suggested wash-out cut-off value of 13.9%, we found that, among the adrenal lesions presenting with a type 2 PE, 16 were mischaracterized as nonadenomas and 33 were correctly characterized as adenomas.

**Table 3:** Diagnostic utility of wash-out evaluation methods.

Evaluation method	AUC (95% CI)	Cut-off	Sensitivity	Specificity
Absolute wash-out				
Type 1 PE	0.735 (0.597–0.846)	38.4%	71.4%	80%
Type 2 PE	0.721 (0.596–0.825)	23.3%	64%	80%
Relative wash-out				
Type 1 PE	0.596 (0.454–0.727)	29.8%	38.8%	100%
Type 2 PE	0.857 (0.748–0.932)	13.9%	68%	100%

## DISCUSSION

CSI is recognized as the cornerstone of the MRI characterization of adrenal lesions^(3-7)^. Our results show that CSI alone had a sensitivity and specificity of 94.4% and 100%, respectively, when an ASII cut-off of 20.4% was applied. However, some benign lesions have no significant lipid content, whereas some nonadenomas display high lipid content, and our findings are in agreement with those of previous reports^(10,11)^. To aid in this characterization, we propose a modified approach to evaluating adrenal lesions when utilizing a standard abdominal MRI protocol. Our study differs from other reports describing contrast enhancement and wash-out of adrenal nodules^(14-17)^ in that we divided adrenal masses into three categories according to the phase during which the wash-in peaked, using a standard post-gadolinium abdominal MRI protocol. Our findings suggest that this categorization provides additional advantages to CSI for the differentiation of adenomas from nonadenomas, especially for lesions with low lipid content or in the presence of extra-adrenal primary tumors that may result in lipid-rich adrenal metastases.

One important observation in our study was that the vast majority of adrenal adenomas (96.1%) showed a type 1 or type 2 PE (47.6% and 48.5%, respectively). In contrast, the majority of nonadenomas showed a type 2 or type 3 PE (38.5% and 48.7%, respectively).

A type 1 PE was found to be strongly associated with a lesion being benign. Our results show that the probability of a lesion with a type 1 PE being an adenoma was 90.9%. Chung et al.^(15)^ and Foti et al.^(20)^ evaluated malignant adrenal lesions and found that none exhibited the peak of enhancement in the arterial phase. However, it is recognized that hypervascular nonadenomas, including pheochromocytoma, metastases from hypervascular extra-adrenal primary malignancies such as renal cell carcinoma or hepatocellular carcinoma, and primary adrenal carcinomas, may show a type 1 PE^(10,11)^. In our study, five nonadenomas (three pheochromocytomas, one adrenal carcinoma and one clear cell renal carcinoma metastasis) showed a type 1 PE. Our results are in keeping with reports suggesting that certain types of adrenal nonadenomas display arterial phase wash-in, rendering them virtually indistinguishable from adenomas based on dynamic contrast imaging^(10,11,21-25)^. A recent study by Choi et al.^(22)^ showed that metastases from renal cell carcinoma and hepatocellular carcinoma can behave similarly to adrenal adenomas in terms of the proportional enhancement and wash-out attained in delayed contrast-enhanced CT studies. We believe that the adoption of an absolute wash-out calculation may aid in differentiation, given that using an absolute wash-out cut-off value of 38.4% provided acceptable sensitivity and specificity (71.4% and 80%, respectively) in our study. Nevertheless, our findings should be regarded with caution because our sample comprised a small number of nonadenomas presenting with a type 1 PE. Our findings suggest that an adrenal mass exhibiting a type 1 PE is very likely to be an adenoma if all of the following criteria are met: the lesion is indeterminate for adenoma based on CSI; there is no clinical or biochemical suspicion of pheochromocytoma; and there is no known history or suspicion of hypervascular extra-adrenal primary tumor.

Another important finding of our study was that type 3 PE was the most common type of PE among the nonadenomas and was quite rare among the adenomas (identified in only 3.9%). Given the relatively low prevalence of lipid-poor adenomas, the probability of a type 3 PE occurring in a lipid-poor adenoma compounds two unlikely events (range, 0.2-0.8%). Therefore, an important observation is that the probability of a lipid-poor lesion with a type 3 PE being a nonadenoma is greater than 99%. In the present study, all adrenal adenomas showing a type 3 PE were, as expected, lipid-rich adenomas. In one case, a metastasis of liposarcoma showed an ASII of 20.4% and a type 3 PE; which, if evaluated by CSI alone, would have been classified as a lipid-rich adenoma^(4,11)^ and considered benign. Our opinion is that the presence of a type 3 PE, independent of the presence of fat on a CSI scan, should raise the suspicion of a nonadenomatous lesion, especially in a patient with a history of a primary extraadrenal neoplasm.

A type 2 PE showed considerable overlap between adenomas and nonadenomas. The wash-out ratio is usually higher for adenomas than for nonadenomas because benign lesions tend to show steeper wash-out slope; that is, greater washout over a shorter time^(1,26,27)^. Our results show that applying a relative wash-out cut-off value of 13.9% provides a clear separation between adenomas and nonadenomas included in the type 2 PE category, with a sensitivity and specificity of 68% and 100%, respectively. Using that cut-off value, both lipid-poor adenomas with a type 2 PE were correctly characterized. We find it interesting that the relative wash-out calculation showed better results than did the absolute washout for type 2 PE and vice versa for type 1 PE. The shorter time window of wash-out obtained with standard protocols, albeit longer for lesions with a type 1 PE and shorter for lesions with a type 2 PE, could explain these differences.

Analogous to previous studies^(25,27)^, the present study demonstrated considerable overlap in size between adenomas and nonadenomas. In our study, the size criterion yielded a relatively low sensitivity and specificity to be accepted as a discriminator per se. Likewise, there were significant differences between adenomas and nonadenomas in terms of the mean wash-in proportion, although the specificity values were less than acceptable because of the considerable degree of overlap.

Our observations are of clinical utility, because the diagnostic accuracy of adrenal nodule evaluation does not depend on the calculation of extensive quantitative data but rather only on the phase during which the peak of enhancement occurred. Our findings support the possibility that the presence of intracytoplasmic lipid identified by means of CSI continues to be the strongest indicator of benignity. In the absence of intracytoplasmic lipid, a lesion presenting with a type 1 PE is very likely to be an adenoma, whereas a lesion presenting with a type 3 PE is very likely to be a nonadenoma. There was considerable overlap for lesions presenting with a type 2 PE, and in our approach we suggest the choice of the relative wash-out calculation as an additional tool to separate adenomas from nonadenomas.

Our study has limitations. First is the fact that it was retrospective. However, this is a circumstance commonly encountered, especially in a preliminary study. Nevertheless, prospective studies could further validate our results and lead to the development of an imaging clinical-decision algorithm integrating CSI and dynamic contrast enhanced imaging. Another limitation is the relatively low prevalence of lipid-poor adenomas in our consecutive population compared with those of previous reports^(3,6,7,12)^. However, we believe that the PE of lipid-poor adenomas should parallel that of lipidrich adenomas, corroborating our results, particularly for the very unlikely event of identifying lipid-poor adenomas with a type 3 PE. In addition, for most of the lesions considered adrenal adenomas, we had no histological confirmation of the diagnosis, which is a limitation of nearly all studies involving imaging of adrenal adenomas. The presence of characteristic imaging features, follow-up stability, and the absence of known extra-adrenal primary neoplasms render biopsy clinically unnecessary and ethically unwarranted.

In conclusion, we proposed a modified approach to the diagnosis of adrenal nodules using a standard abdominal MRI protocol. Characteristic findings on CSI continue to represent the strongest indicator of benignity. However, the categorization of lesions based on dynamic PEs may yield high diagnostic accuracy in the differentiation of adenomas from nonadenomas when diagnosis by CSI is not possible.
